# Acute versus chronic mitral regurgitation: a case of perioperative cardiogenic shock in an athlete

**DOI:** 10.1093/jscr/rjaf1089

**Published:** 2026-02-27

**Authors:** Ross Walsh, Jamie Walsh, Robert Doyle, Laura Casey, Hossein Javadpour

**Affiliations:** Royal College of Surgeons of Ireland, 123 St Stephen's Green, Dublin 2 D02 YN77, Ireland; Royal College of Surgeons of Ireland, 123 St Stephen's Green, Dublin 2 D02 YN77, Ireland; Department of Cardiothoracic Surgery, The Mater Misericordiae University Hospital, Dublin, Ireland; Department of Cardiothoracic Surgery, The Mater Misericordiae University Hospital, Dublin, Ireland; Department of Cardiothoracic Surgery, The Mater Misericordiae University Hospital, Dublin, Ireland

**Keywords:** mitral regurgitation, flail leaflet, cardiogenic shock, VA-ECMO, perioperative instability, athlete heart

## Abstract

Mitral regurgitation (MR) poses challenges in distinguishing acute from chronic aetiologies in compensated patients. A 48-year-old male rugby player presented with 2 weeks of worsening dyspnoea on a background of chronic bilateral lower limb cellulitis. Found in atrial fibrillation, transoesophageal echocardiography revealed flail P2 with severe MR, ejection fraction 35%, and severe left ventricular dilation—indicating chronicity despite acute symptoms. He underwent urgent mitral valve repair. Post-induction instability necessitated emergency cardiopulmonary bypass. Repair consisted of P2 triangular resection, neochordae, and Cosgrove annuloplasty band. Weaning failed; central veno-arterial extracorporeal membrane oxygenation was instituted with open chest. Decannulated day 4, complications included anuric acute kidney injury requiring dialysis, pneumonia, and critical illness myopathy. Ejection fraction improved to 30% on medical therapy. This case underscores risks of presuming acute MR in athletes with chronic remodelling, where perioperative decompensation can precipitate shock. Preoperative haemodynamic optimization is critical to mitigate such events.

## Introduction

Severe mitral regurgitation (MR) due to posterior leaflet prolapse is typically amenable to repair with excellent outcomes [1]. However, distinguishing acute from chronic MR remains challenging, particularly in physically active individuals with robust compensatory mechanisms. Chronic severe MR may remain asymptomatic for years due to preload augmentation and eccentric hypertrophy, only decompensating under stress [2]. This case illustrates perioperative failure in a fit 48-year-old athlete with presumed acute severe MR, ultimately revealing evidence of chronic volume overload.

## Case presentation

A 48-year-old male rugby player presented with progressive dyspnoea and palpitations. Symptoms had worsened over 2 weeks, including exertional shortness of breath, orthopnoea, lethargy, and episodic palpitations. He denied chest pain, or fever. He had history of bilateral leg oedema and recurrent cellulitis for over a decade but had no prior cardiac history. He was on no regular medications.

**Figure 1:**
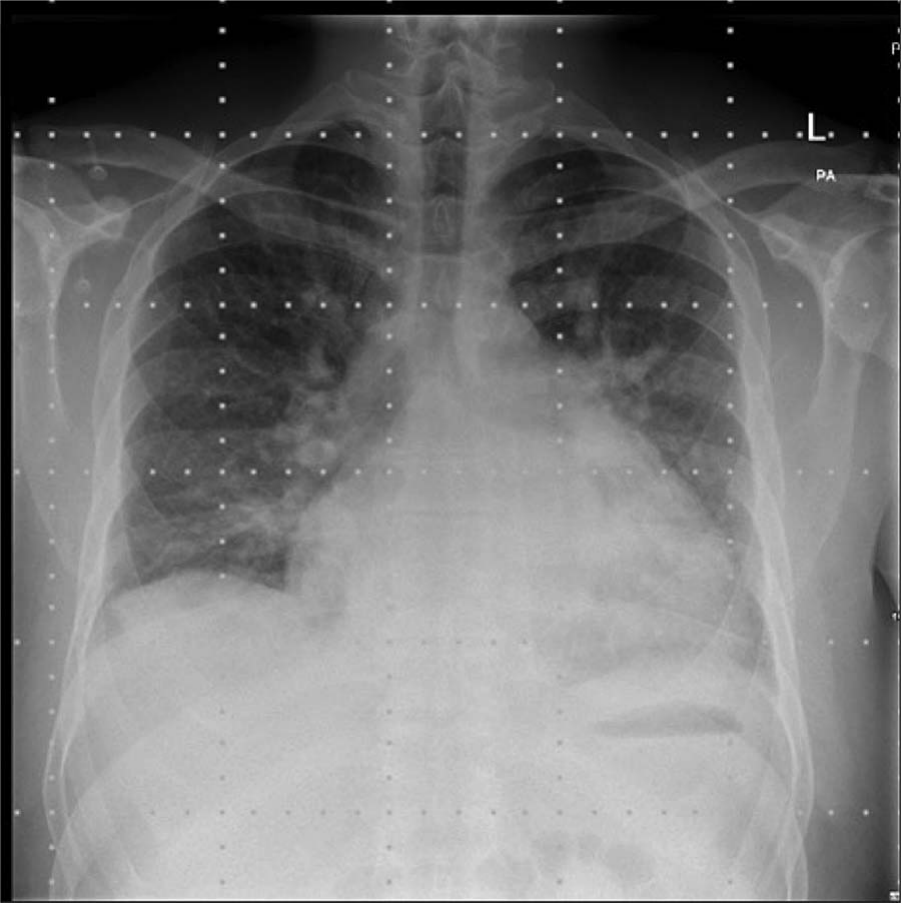
Pre-operative chest radiograph.

**Figure 2:**
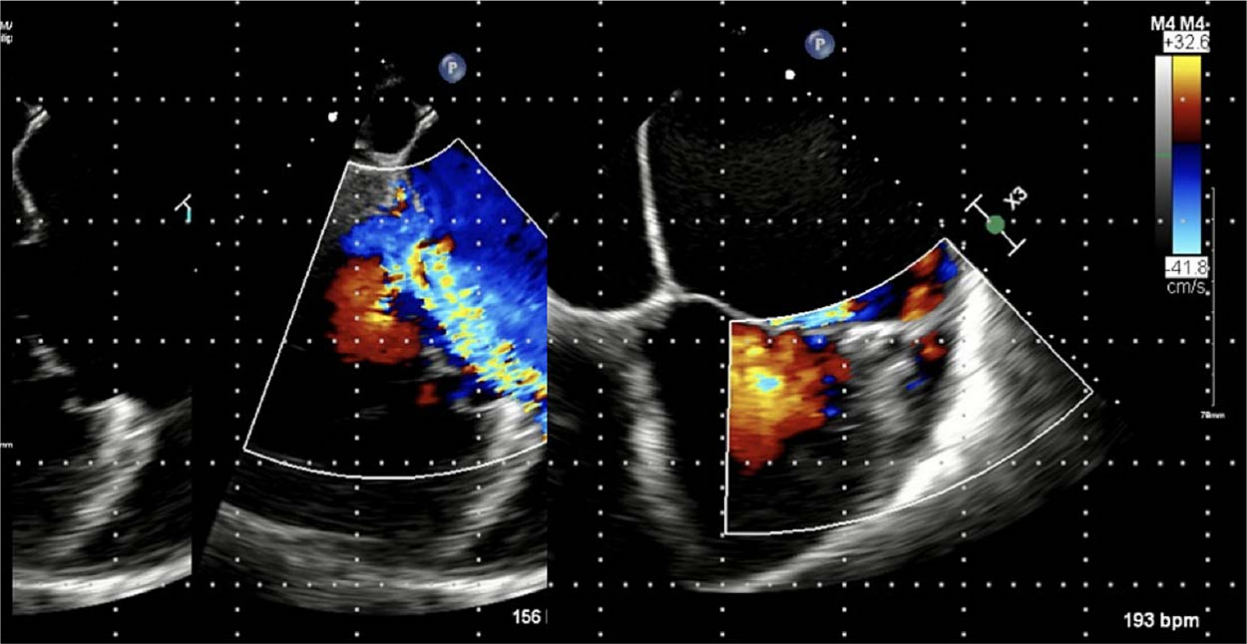
Pre-operative trans-oesophageal echocardiogram (TOE).

Chest radiograph demonstrated pulmonary oedema (Fig. 1) and transoesophageal echocardiography (TOE) demonstrated a flail P2 segment with an anteriorly directed jet of severe MR, vena contracta 8 mm, effective regurgitant orifice area 0.55 cm^2^ (Fig. 2). Left ventricle was severely dilated (LVEDD 72 mm) with ejection fraction 35%. Both atria and the left ventricle were dilated. No vegetations were seen. He was cardioverted to sinus rhythm with 200 J biphasic shock.

Coronary angiography showed normal left main stem, left anterior descending, and circumflex arteries; the right coronary artery had 40% ostial stenosis. Following Heart Team discussion, urgent mitral valve repair was decided. Despite severe bi-atrial and left ventricular enlargement, the consensus was that this was acute MR, given the chronicity of symptoms and the excellent pre-morbid baseline.

He was transferred to our tertiary referral centre for surgical repair. After induction of anaesthesia, the patient developed atrial fibrillation with rapid ventricular response (>160 bpm), hypoxia (SpO₂ 82%), and hypotension (60/40 mmHg). TOE showed acute biventricular dilation and failure. Emergency cardiopulmonary bypass (CPB) was instituted via aortic and bi-caval cannulation.

The mitral valve was exposed via left atriotomy. A flail P2 segment was confirmed with ruptured primary chordae. Repair included triangular resection of P2, implantation of two pairs of Gore-Tex neo-chordae to P2 remnants, and a 38 mm Cosgrove annuloplasty band. The left atrial appendage was amputated. CPB time was 3 hours 25 minutes; aortic cross-clamp time 1 hour 58 minutes.

Weaning from CPB was unsuccessful despite maximal inotropic support. Post-repair TOE showed EF 10%–15% with global hypokinesis. Central veno-arterial extracorporeal membrane oxygenation (VA-ECMO) was established via right atrial inflow and ascending aortic outflow at 6.5 L/min. The chest was left open and patient transferred to ICU. Patient was coagulopathic with requiring 10 units packed red cells, 22 units fresh frozen plasma, 5 platelet pools and 15 g fibrinogen concentrate. Anuric acute kidney injury necessitated continuous renal replacement therapy (CRRT) from postoperative day 1.

Patient was stabilized over the following days on ECMO with reducing inotropic support. He returned to theatre Day 3 for mediastinal haematoma evacuation. ECMO weaning study showed stable mean arterial pressure at reduced 0.5 L/min flow and on post-operative day 4, the patient returned to theatre for ECMO decannulation with intra-aortic balloon pump (IABP) insertion. This was subsequently removed on Day 7.

Initial extubation on Day 9 failed due to respiratory secretions. Re-intubation and bronchoscopy (for clearance of mucous plugging of the left main bronchus) was required. He was successfully re-extubated on Day 14. Hospital-acquired pneumonia was treated with co-trimoxazole. Critical illness myopathy delayed mobilization. CRRT was transitioned to intermittent haemodialysis on Day 20.

Follow-up transthoracic echocardiography showed improved LV function (EF 30%) with no residual MR and persistent biventricular dilation. Guideline-directed medical therapy was commenced and up titrated. He remains as in patient undergoing ongoing rehabilitation at time of writing.

## Discussion

This case highlights the diagnostic and therapeutic pitfalls of severe MR in a compensated athlete. The acute presentation with flail leaflet suggested primary chordal rupture, yet echocardiographic findings—severe LV dilation, bi-atrial enlargement, and reduced EF—indicated chronic volume overload [3]. Athletic individuals may tolerate severe MR for decades due to enhanced diastolic compliance and preload reserve, masking chronicity until acute triggers (e.g. AF, infection) precipitate decompensation [4]. Similar silent progression has been documented in athletes, where high functional capacity delays symptom onset despite significant remodelling [5].

Perioperative collapse stemmed from anaesthetic induction, which acutely reduced preload and systemic vascular resistance, thereby increasing the regurgitant fraction and precipitating biventricular failure in a chronically volume-overloaded heart. Difficulty weaning from CPB was exacerbated, ironically, by the surgical correction putting more pressure on the decompensated ventricle. This "afterload mismatch" phenomenon is well-described in chronic MR, where sudden correction eliminates the regurgitant pathway, stressing a ventricle adapted to low-resistance ejection [6].

While early surgery is recommended for severe MR with flail leaflet [7], this case suggests caution in patients with evidence of chronic remodelling. Preoperative optimization—diuresis, afterload reduction, and rhythm control may stabilize haemodynamics [8]. Temporary mechanical circulatory support (MCS) (e.g. Impella, IABP) prior to induction could bridge high-risk patients, as prophylactic MCS has reduced mortality in similar high-risk valve cases [9].

VA-ECMO provided lifesaving biventricular unloading and oxygenation, allowing myocardial recovery. Despite initial EF 10%–15%, LV function improved to 30% within a few weeks, supporting the role of mechanical unloading in reversible cardiogenic shock [10]. ECMO outcomes have improved with central cannulation and open-chest strategies, as seen in our approach [11]. Multi-organ support, including CRRT for acute kidney injury (AKI), was crucial, aligning with Extracorporeal Life Support Organization guidelines [12].

Long-term, guideline-directed therapy targeting neurohormonal activation is essential for reverse remodelling in recovered patients [13, 14]. This case adds to the literature on MR acuity misjudgement, emphasizing integrated echocardiographic quantitation and careful operative planning.

## Conclusion

Presumptive acute MR in a fit patient with chronic echocardiographic changes carries high perioperative risk. Surgical repair remains definitive, but preoperative haemodynamic optimization and readiness for mechanical circulatory support are essential. This case reinforces the need for multidisciplinary assessment and tailored perioperative strategies in complex MR.

### Learning points

1. Chronic severe MR may be asymptomatic in athletes until acute decompensation.
2. Echocardiographic evidence of chamber dilation and reduced EF suggests chronicity despite acute presentation.
3. Anaesthetic induction in severe MR risks acute afterload reduction and biventricular failure.
4. VA-ECMO is effective salvage therapy in postoperative cardiogenic shock with potential for myocardial recovery.
